# Clear Conversations: a mixed methods evaluation of a verbal health literacy initiative for health service providers

**DOI:** 10.1186/s12913-026-14684-y

**Published:** 2026-05-09

**Authors:** Cheryl Grindell, Jo Hall, Laura Connolly, Jane Hawley, Alicia O’Cathain

**Affiliations:** 1 Sheffield Centre for Health and Related Research (SCHARR), Division of Population Health, The University of Sheffield, Regents Court, Sheffield, S1 2DA UK; 2 Derbyshire Community Health Services NHS Trust, Ashgate Road, Chesterfield, S42 7JE UK; 3 Derbyshire County Council, County Hall, Matlock, DE4 3AG UK

**Keywords:** Verbal health literacy, Implementation support, Health and wellbeing outcomes

## Abstract

**Background:**

Understanding health information can be difficult, which can limit people’s ability to manage their health and adopt healthy practices, leading to poorer health outcomes. This study aimed to evaluate a verbal health literacy training initiative designed to support clear conversations between health service providers and users. The initiative incorporated Teach-back, Chunk and Check, Simple Language, and Open Questions, alongside implementation support from a health literacy officer.

**Methods:**

A concurrent mixed-methods evaluation was conducted across two health programmes serving a population of approximately one million people in the United Kingdom: A Pulmonary Rehabilitation Programme delivered by five physiotherapists in a hospital setting, and a Weight Management Programme delivered by 12 health improvement advisors within a regional government authority. The evaluation comprised five components: (1) Surveys of 110 service providers’ perceptions of the training; (2) Observations of 11 service providers before and after training; (3) Two focus groups with 11 service providers six months post-training; (4) Change in 73 service users’ health literacy levels using two domains of the Health Literacy Questionnaire; (5) Change in service users’ health and wellbeing between baseline and programme completion.

**Results:**

Service providers found the initiative useful. Changes in communication practice, such as increased use of Chunk and Check and Open Questions, were observed. Both programmes were delivered in group settings. Teach-back was reported to be challenging to apply in this context but beneficial in one-to-one interactions in wider practice. Implementation support from the health literacy officer was helpful but difficult to deliver to busy teams. Service users’ health literacy levels improved by the end of their programme, but there was no evidence that the initiative improved health and wellbeing outcomes. For example, no significant difference was observed in the primary outcome for Weight Management participants in the intervention group compared with controls (0.2 kg, − 2.3 to 1.9; *p* = 0.84).

**Conclusions:**

In this small evaluation, the initiative was well received and enhanced service providers’ communication skills. Further evaluation of a strengthened initiative should focus on controlled before-and-after designs using larger samples to determine the effect on service users’ health literacy and health and wellbeing outcomes.

**Supplementary Information:**

The online version contains supplementary material available at 10.1186/s12913-026-14684-y.

## Background

Health literacy is important so that everyone has the ability to access and receive high quality healthcare [1]. Health literacy has been defined as a person’s ability to understand and use information to make decisions about their health so they can be active partners in their care [2]. People with low health literacy may struggle to read and understand health information, communicate with health professionals, and know how to act on the information they receive [3]. If health literacy is improved this can positively affect treatment adherence, and lead to reduced numbers of cancelled appointments, and improved satisfaction and outcomes [4]. Organisational health literacy is about how healthcare institutions help service users to access, understand, and use information and services to manage their health [5]. It shifts responsibility from the service user to the service provider. Improving healthcare service providers’ communication skills is an important part of this and has the potential to positively impact on service users [5–9]. Evidence suggests that effective health conversations can potentially increase adherence to treatment, patient safety, quality of life and health outcomes [5–7].

In the United Kingdom (UK) health care is provided by the National Health Service (NHS) free at the point of delivery. National agencies advocate the use of clear communication, both spoken and written, to help address health literacy [10, 11]. These agencies have developed toolkits and guides where they recommend the use of: Teach-back techniques [10, 11], a method to ensure patients have understood the health information they receive by asking them to explain what they have been told to do [11]; Chunk and Check techniques [10, 11], where information is broken down into smaller chunks rather than giving it all at once, then checking it is understood before moving onto the next ‘chunk’[11]; Simple Language, that avoids the use of jargon and acronyms [10]; and Open Questions that cannot be answered with a ‘yes’ or ‘no’. For example, ‘what questions do you have’, in order to invite and elicit questions from service users [12]. Verbal health literacy techniques such as these have been evaluated internationally [7, 8, 13–18]. Evidence suggests that Teach-back, can improve knowledge recall, reduce hospital readmissions, and enhance quality of life [18] and in long term condition management it may support self-management and self-efficacy [15–17]. However, findings on knowledge retention are inconsistent and there is limited evidence on how these approaches are embedded into routine practice [18].

Training alone has been found to be insufficient for sustained implementation of verbal health literacy techniques in practice [14, 18]. A study in the UK, where pharmacists received a one-off training session on Teach-back, Chunk and Check, and Simple Language, highlighted practical challenges with some techniques being harder to apply and a tendency to revert to jargon [19]. Observational studies have also been recommended to validate healthcare providers self-reported improvements after receiving communication training [20]. The organisational health literacy literature emphasises the limited understanding of how well health literacy techniques are implemented in practice [5]. This highlights the need for additional implementation support such as health literacy champions or facilitators [20, 21].

When we designed this study in October 2023, the evidence base for how to train service providers to consistently apply verbal health literacy techniques in order to improve service users’ health and wellbeing outcomes was limited. Therefore, there was a need to evaluate initiatives to improve health service providers’ communication skills, particularly measuring whether such initiatives improve service users’ health and wellbeing outcomes in the short and longer term. The aim of this study was to evaluate a verbal health literacy initiative offered to different types of service providers aiming to improve service users’ health and wellbeing.

## Methods

### Setting

Derbyshire is a region of the UK with a population of a million people. Six out of ten people are estimated to be below the UK average for health literacy and numeracy [22]. ‘Joined Up Care Derbyshire’ brings together services provided by the NHS, regional government authorities (known as local authorities), and the voluntary sector to deliver better care to whole communities in Derbyshire [23]. In 2022 the regional agency ‘Joined Up Care Derbyshire’ appointed a health literacy officer. This is a role that is not commonly provided in the UK. The health literacy officer offers support and training to service providers to improve health literacy within their services.

### The programmes delivering services

Two programmes were selected to participate in the evaluation. First, the Pulmonary Rehabilitation Programme provided within the NHS, which aims to improve patients’ exercise tolerance and respiratory health. This programme provides a six week rolling face-to-face programme for adults aged 18 and over. Fifteen members of staff are involved in the delivery of the programme. The staff include a mixture of physiotherapists, healthcare assistants and specialist nurses. They offer supervised exercise, education and advice to groups of people who are limited by their respiratory condition. Five physiotherapists lead the group sessions. Up to 12 service users attend each session twice a week over the six week period. Second, Live Life Better Derbyshire’s Tier 2 Weight Management Programme. This programme aims to help adults aged 18 or over lose weight. The service provides online and face-to-face group education and advice programmes that run over a 12 week rolling period. Twelve Health Improvement Advisors lead the group education and advice sessions. Up to 25 people, who wish to lose weight, attend each session once a week over the 12 week period.

### The verbal health literacy initiative

The verbal health literacy initiative is part of a wider initiative called ‘Quality Conversations’. This is an evidence-based communications skills programme for health service providers. It has been developed by the Derbyshire Psychological Insights team and Public Health Derbyshire, to support the implementation of ‘Making Every Contact Count’ in Derbyshire [24]. Quality Conversations is informed by the COM-B behaviour change model [25]. COM-B is an evidence-based framework stating that behaviour occurs through the interaction of three components: Capability (physical/psychological skills); Opportunity (physical/social environment); and Motivation (conscious/unconscious, cognitive processes) [25]. Quality Conversations ensures that staff have the skills to facilitate health literacy (capability), are made aware of where they can facilitate health literacy (opportunity) and understand the potential benefits of facilitating health literacy (motivation). Quality Conversations focuses on addressing health inequalities through the promotion of ‘*compassionate and curious conversations’* [24]. A health literacy officer works with teams to support them to take evidence-based steps to provide more health literacy friendly services.

The initiative consists of a generic verbal health literacy training package for health service providers to enable clearer health conversations. The training is a two-hour interactive and experiential session delivered by the health literacy officer. It focuses on how service providers can learn, practice and implement four key verbal health literacy techniques described earlier: Teach-back, Chunk and Check, the use of Simple Language, and Open Questions. The health literacy officer then offers service providers tailored implementation support to help them use the techniques within their practice. This might consist of a meeting with teams to discuss any issues they may be having implementing the techniques and providing advice on how to overcome them. Please see supplementary file 1 for a breakdown of the training schedule.

All five physiotherapists from the Pulmonary Rehabilitation Programme received the training because they are the team members that spend most time with service users. Half of the health improvement advisors from the Weight Management Programme (6/12) received the training to allow for a controlled before and after comparison of outcomes for this service. These physiotherapists and health improvement advisors are referred to as service providers. The 11 service providers who participated in this study received the Verbal Health Literacy training initiative in July 2024.

### Study design

A concurrent mixed methods evaluation was undertaken consisting of a process evaluation to assess acceptability, feasibility and implementation of the initiative [26], and a concurrent pre-test post-test measurement of outcomes [27, 28].

### The research team

The research team consisted of the head of the Derbyshire Psychological Insights team who designed the initiative, the health literacy officer who designed and provides the initiative, a public health practitioner from Derbyshire County Council who was involved in the development and delivery of the initiative, and two university researchers (one of whom is an academic physiotherapist (CG)).

### Ethics

Ethics approval was given by Camden and Kings Cross NHS Research Ethics committee (REC reference: 24/LOC/0324).

### Patient and public involvement and engagement

Seven service users were recruited from the services and existing patient and public panels to form a project partnership advisory group. The group met online three times to help develop plain English versions of service user research documents and provide advice about recruitment, analysis and interpretation of results. Payment was offered based on national guidance for public contributors’ payment recommendations [29].

### Proposed theory of change

**Fig. 1:**
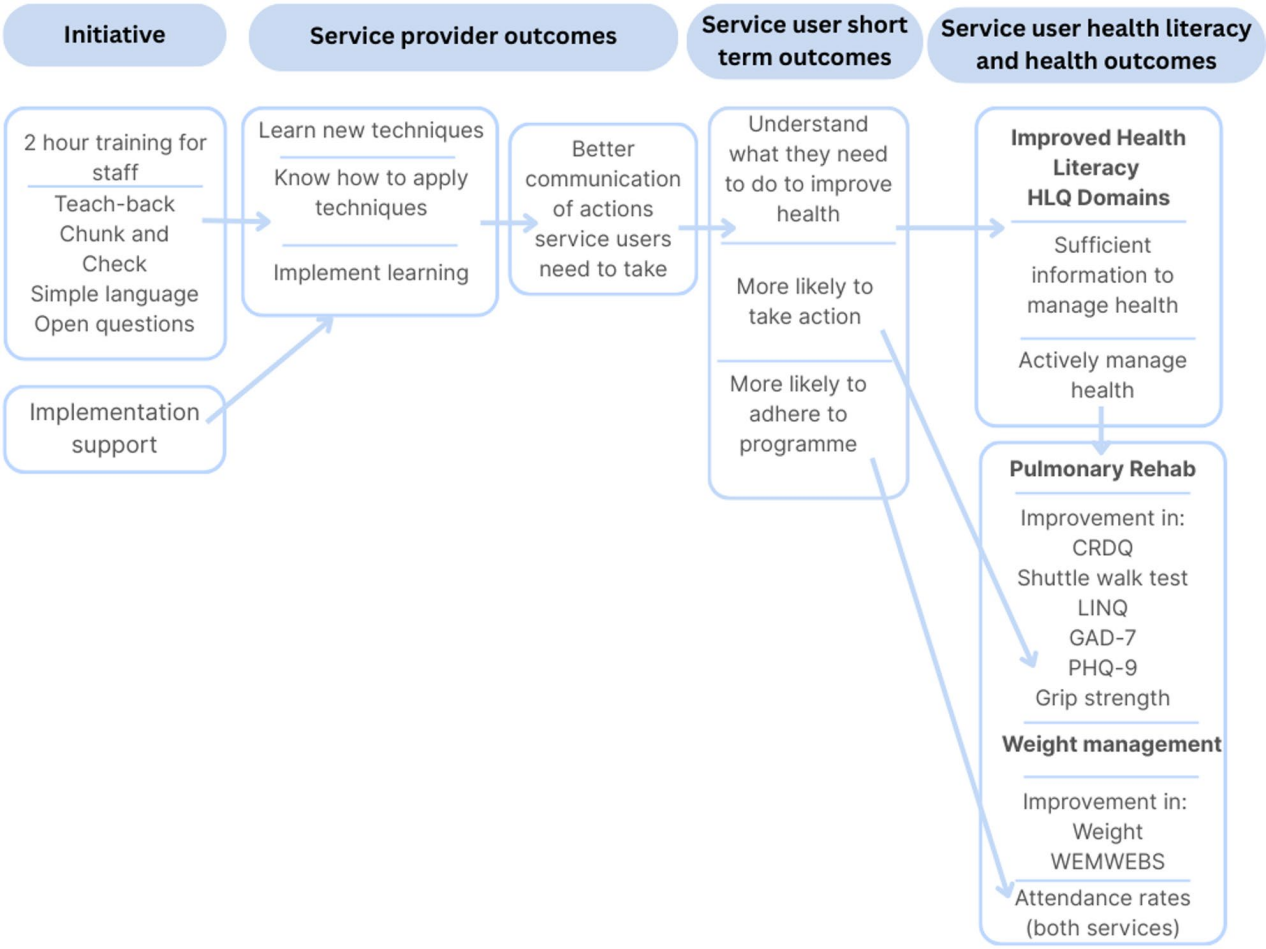
Theory of change for the verbal health literacy initiative

COM-B was not used in the evaluation. The research team developed a theory of change for the initiative (see Fig. 1). The hypothesis was that: service providers would learn new techniques; leading to clearer conversations with service users; who would then understand what actions they needed to take to improve their health; which would then lead to improved service user health outcomes.

### Components of the evaluation

There were five components of the evaluation. Please see supplementary file 2 for a timeline of the evaluation and its components.

#### Survey of service providers’ views of the initiative

As part of routine practice the health literacy officer undertook an online survey of attendees at the training session at three time points: at baseline where current knowledge of verbal health literacy techniques was assessed; immediately after training where the utility of the training and knowledge and confidence to use the techniques was assessed; and at 12–20 weeks post-training where confidence, opportunity and frequency of use of the techniques, along with support for implementation, was assessed. These pre-existing surveys that were developed and already being used by the health literacy officer were used for the study. See supplementary files 3, 4 and 5. Anonymised data on all service providers undergoing training in a 26 week period (July 2024 - January 2025) were provided to the research team. This data included the 11 service providers in this evaluation. Data was only included in this research if individual consent was given.

#### Observation of service delivery before and after training

The lead researcher (CG) observed two consultations with service users for each of the 11 service providers in the evaluation. This was to assess if there was a change in the frequency with which service providers used the techniques they learnt. One consultation was observed before training, and one four to six months after training. The lead researcher recorded the extent to which the techniques (Teach-back, Chunk and Check, Simple Language and Open Questions) occurred in each consultation on a seven-item checklist using the response set of ‘never, sometimes, most of the time, all of the time’.

#### Focus groups of service providers’ views of the initiative

Two focus groups were undertaken with the 11 service providers in the evaluation who received training [30]. A focus group was undertaken for each programme to allow discussion of issues important to their area of practice. The focus group took place online for the five physiotherapists from the Pulmonary Rehabilitation Programme. The focus group took place face to face for the six health improvement advisors from the Weight Management Programme. The focus groups were facilitated by CG. They lasted 90 min and were recorded and transcribed.

#### Survey of change in service users’ health literacy levels

Service users, who attended the two programmes in the period after the service providers were trained, were consented to participate in the evaluation. This meant that data were available in the post-test period only. Changes could not be assessed between service users receiving programmes before the verbal health literacy training and after the training. For the Pulmonary Rehabilitation Programme, physiotherapists approached service users to request sharing their contact details with the lead researcher. The lead researcher then contacted service users for written informed consent. For the Weight Management Programme, written consent was taken via an electronic link when service users enrolled on the programme.

If service users gave consent, they completed a questionnaire which included two domains, out of the nine domains of the validated Health Literacy Questionnaire [3]. Several of the domains were relevant but only two domains of the Health Literacy questionnaire were included due to concerns about research burden on service users. The team selected the two most relevant domains: whether they felt they had sufficient information to manage their health, and whether they felt they were actively managing their health. Service users were asked to consider the questions in relation to the programme they were about to, or had, attended. They completed the questionnaire electronically or by telephone with the lead researcher. The domains were completed at two time points: baseline (before they attended the programme) and at the end of their six weeks (Pulmonary Rehabilitation) or 12 weeks (Weight Management) programme.

#### Routine data to assess health and wellbeing outcomes

##### Individual service user data

The two programmes collect data about service user outcomes as part of their routine service. Data for individual service users was shared with the researchers for service users who gave written informed consent (see section above). For the Pulmonary Rehabilitation Programme, assessments were carried out at baseline and on completion of the programme at six weeks, after attendance at eight to 12 sessions. The primary outcome was change in quality of life measured by the four domains of the Chronic Respiratory Disease Questionnaire (CRDQ) [31]. Secondary outcomes were measured using the incremental shuttle walking test in metres [32], Lung Information Needs Questionnaire (LINQ) [33], Generalised Anxiety Disorder Assessment (GAD − 7) [34], Patient Health Questionnaire 9 (PHQ-9) [35] and strength tests at 0 and 6 weeks [36]. For the Weight Management Programme, service users completed routine service assessments at baseline, 12 and 26 weeks. The primary outcome was change in weight in kilograms. Weight was self-reported for those attending the programme online. Those attending the programme face-to-face were weighed by the service providers weekly. Due to time constraints for the research, the primary outcome was weight change between 0 and 12 weeks only. The secondary outcome of well-being was measured by the short form Warwick and Edinburgh Mental Health and Wellbeing Scale [37] at 0 and 12 weeks.

##### Programme level data

Assessment of aggregated programme level data from each of the two programmes was important. This was because the individual level data required informed consent from service users, and this was likely to achieve only partial sign up. The Pulmonary Rehabilitation Programme provided data for the six month period after the training (July 2024-December 2024), and for the same six month period in the year before the training (July 2023-December 2023). This was to help reduce seasonal effects. The Weight Management Programme leaders had recently made changes to the programme. They felt these changes would adversely affect a similar analysis. They therefore agreed to provide data for a three-month period post-training (September -November 2024), and the three month period immediately before training (May-July 2024). The data was attendance numbers, programme completion numbers, and outcomes described in the individual level outcomes section earlier. This data was presented in terms of means and standard deviations for the periods pre- and post-training. For the Weight Management Programme, the data was divided into courses led by the six verbal health literacy trained service providers and a control group of courses led by six service providers who had not attended the training. Service providers were not randomised to attend training but were selected by their manager to do this. A before and after comparison was made of the data from the intervention and control groups.

##### Expected sample size for outcomes

For the Pulmonary Rehabilitation Programme, the research team estimated that 96–144 service users might enrol in the post-training period. With an expected response rate of 50% for giving consent, individual outcomes were expected for around 70 service users in the post-test period. Data was not available to the research team to undertake a formal sample size calculation. For the Weight Management Programme, the research team estimated that 495–825 service users might enrol in the post-training period. With a response rate of 50% for giving consent, individual outcomes were expected for 330 service users. Approximately 165 service users would have attended courses led by trained service providers and 165 service users would have attended courses led by providers who had not attended the training. A sample size calculation was undertaken using data from 2023 where the mean starting weight was 103.68 kg. The mean weight on completion of the course was 99.60 kg. So, the mean weight loss was 4.08 kg for this cohort. If the initiative resulted in an extra 10% increase in mean weight loss at 12 weeks, with an estimated pooled standard deviation of 1.6, a sample size of 252 would be required in each group to detect this size of difference with 80% power at a 5% level of significance.

### Data analysis

The lead researcher entered data from the service provider surveys into SPSS [38]. They calculated descriptive statistics at each of the three time points e.g. how staff rated their knowledge of the techniques (baseline, post-training and at follow up), percentage of staff who found the training helpful (post-training), and percentage of staff who used the techniques in practice (two to four months post-training).

The lead researcher allocated scores to the observations of the 11 service providers’ use of the verbal health literacy techniques in practice. The scores related to the categories in the response set (‘all of the time’= 3 to ‘never’= 0). The lead researcher calculated a total score for each service provider before and after training and entered data into SPSS [38]. They undertook a paired t-test to measure change within individual service providers between pre- and post-training.

For the two focus groups, the lead researcher transcribed the recordings and analysed the two transcripts based on the ‘framework approach’. They followed the four stages of familiarisation, identification of a thematic framework, charting (coding the data to the thematic framework), and mapping (making connections between themes) [39]. They worked closely with another team member who read the transcripts and discussed the themes. The evolving findings were discussed with the wider team during the analysis process.

For the Health Literacy Questionnaire [3] for service users, the lead researcher entered the data into SPSS [38]. They calculated scores for the two domains of the Health Literacy Questionnaire at baseline and the end of the course. The maximum scores for each domain was five. The data were not normally distributed. The lead researcher and another team member worked together to calculate the mean change over time and undertook a paired t-test. T-tests are robust to non-normal distributions for large samples, but the team also undertook Wilcoxen tests which gave the same results. For the Weight Management Programme, there was an intervention group and a control group, so the research team compared changes in health literacy scores for these two groups using a t-test.

For the individual level outcomes, the lead researcher entered the data into SPSS [38] and calculated descriptive statistics for each outcome measure. The team compared mean change over time using a paired t-test for the Pulmonary Rehabilitation Programme. They assessed the difference in mean change over time between intervention and controls using a t-test for the Weight Management Programme.

For the aggregated data, the team calculated the mean change in an outcome in the pre-test and in the post-test periods and compared these using a t-test. For the Weight Management Programme, the team assessed the difference in mean change over time between intervention and controls using a t-test.

## Results

### Participants

All five physiotherapists from the Pulmonary Rehabilitation Programme received the training and participated in the evaluation. Six of the 12 Weight Management Programme advisors received the training, with six acting as controls. The six receiving the training participated in the evaluation. All 11 service providers (five physiotherapists and six health improvement advisors) participated in the observation and the focus groups.

In total 110 service providers completed the pre-training survey, including the 11 who were part of the evaluation. Participation decreased to 69 service users in the post-training survey. This included the 11 service providers who were part of the evaluation. Only 23 completed the final survey, including seven of the 11 in the evaluation (three from the Pulmonary Rehabilitation Programme, four from the Weight Management Programme).

For the Pulmonary Rehabilitation Programme, 49 service users who attended the programme consented to take part: 45% (22/49) male, 96% white (47/49), mean age 69 (range 46 to 85), 6% (3/49) in the quintile with the worst social deprivation using the Index of Multiple Deprivation [40]. This was lower than the expected 70 participants. For the Weight Management Programme 34 service users consented to take part: 18% (6/34) male, 94% (32/34) white, mean age 52, and 21% (7/34) in the quintile with the worst social deprivation. This was lower than the expected 160 participants. Therefore, the analysis of the service user health and wellbeing outcomes lacked statistical power.

### Service providers’ views of the initiative

Two thirds of the service providers (68%, 47/69) found the verbal health literacy training to be very useful and 26% (18/69) useful. For the 11 in the evaluation, 100% (5/5) of the Pulmonary Rehabilitation Programme team found the training to be very useful compared to 33% (2/6) of the Weight Management Programme team. See supplementary file 6 for details.

Most service providers rated their knowledge of the verbal health literacy techniques to be poor to adequate pre-training, especially for the use of Teach-back and Chunk and Check. That is, 82% (90/110) and 74% (82/110) indicated their knowledge was poor, less than adequate or adequate for Teach-back and Chunk and Check respectively. Their knowledge improved post-training and was sustained at four- six months post-training, although numbers were much smaller at this time point. This pattern was evident for the two sets of service providers in the evaluation (see Table 1).

**Table 1:** Service providers’ reported knowledge of the verbal health literacy techniques

Question	All service providers %			Service providers in our study %			Pulmonary Rehab Programme service providers %			Weight Management Programme service providers %		
	*n* = 110	*n* = 68	*n* = 23	*n* = 11	*n* = 11	*n* = 7	*n* = 5	*n* = 5	*n* = 3	*n* = 6	*n* = 6	*n* = 4
On a scale of 1–5 (1 Poor – 5 Excellent) how would you rate your knowledge of:	**Pre**	**Post**	**Follow Up**	**Pre**	**Post**	**Follow Up**	**Pre**	**Post**	**Follow Up**	**Pre**	**Post**	**Follow Up**
**The use of Teach-back** 1 Poor 2 Less than adequate 3 Adequate 4 Good 5 Excellent	14.5 30.0 37.3 17.3 0.9	0 1.4 11.6 52.2 34.8	0 0 21.7 60.9 17.4	27.3 27.3 45.5 0 0	0 0 0 90.9 9.1	0 0 14.3 85.7 0	20 20 60 0 0	0 0 0 100 0	0 0 33.3 66.7 0	33.3 33.3 33.3 0 0	0 0 0 83.3 16.7	0 0 0 100 0
**Chunk and Check** 1 Poor 2 Less than adequate 3 Adequate 4 Good 5 Excellent	10.9 24.5 38.7 24.3 0.9	0 1.4 17.4 47.8 33.3	0 0 13.0 65.2 21.7	36.4 27.3 36.4 0 0	0 0 9.1 81.8 9.1	0 0 14.3 85.7 0	40 20 40 0 0	0 0 0 100 0	0 0 0 100 0	33.3 33.3 33.3 0 0	0 0 16.7 66.7 16.7	0 0 25 75 0
**Simple language** 1 Poor 2 Less than adequate 3 Adequate 4 Good 5 Excellent	6.4 12.7 36.4 40.9 3.6	0 0 10.3 55.9 33.8	0 0 13.0 60.9 26.1	9.1 9.1 45.5 36.4 0	0 0 0 72.7 27.3	0 0 0 100 0	0 0 20 80 0	0 0 0 80 20	0 0 0 100 0	16.7 16.7 66.7 0 0	0 0 0 66.7 33.3	0 0 0 100 0

*Use of Open Questions was not included in the staff questionnaire designed and distributed by the health literacy officer

A similar pattern was seen for how service providers rated their confidence to use and how often they used the verbal health literacy techniques in practice. See supplementary files 7 and 8.

### Observed use of the techniques before and after training

The observation of sessions delivered to service users demonstrated more frequent use of verbal health literacy techniques post-training than pre-training. The maximum score on the observation checklist was 21. The mean score for the 11 service providers in the evaluation increased from 4.5 at pre-training to 9.4 at post-training (*p* < 0.001). The improvements were similar for each programme: 4.4 to 9.0 (*p* = 0.019) for the Pulmonary Rehabilitation Programme and 4.5 to 9.7 (*p* < 0.001) for the Weight Management Programme. Some verbal health literacy techniques were not used at all in the pre-training period (Chunk and Check) but were used sometimes in the post-training period.

### Service providers’ views of the initiative

Four themes were developed from the focus group data relating to: the training (relevant and worthwhile), the ability to make changes to materials they used (varied ability), implementing the techniques in routine practice (limitations of the group setting), and the post-training implementation support (more needed).

### The training was relevant and worthwhile

Overall, the training was received favourably by both teams. All the physiotherapists and the majority of the health improvement advisors described how they found the training enjoyable, relevant, and worthwhile. They liked the varied and interactive content which they found engaging. They felt that it should be offered to all staff and used in undergraduate level education. They suggested minor changes to the training such as more time to practice the techniques and tailoring the techniques for use in group settings and specific scenarios.

*Other health professionals teaching the [courses]*,* they could all do with this training*,* couldn’t they? I’d advise anyone to go on it that hasn’t done the training that’s in health care*,* definitely. We could have done with it at university couldn’t we?* (Physiotherapist 4)

*I’d ask for more group-based examples. I think it felt like the training was set up for one-to-one situations perhaps more than groups a little bit more. Or maybe just if there’s time to get to know some of the nuances of the service and the practice of the professionals to be able to get really useful specific examples that are hyper-appropriate*,* that would be what I would add to it*. (Health Improvement Advisor 10)

### Ability to make changes to materials used with service users

Both teams reported they were actively trying to use the techniques in their practice. The physiotherapists described being unfamiliar with the techniques and did not use them at all prior to the training. They had changed the PowerPoint slides they used with service users as a result of the training to help them incorporate the verbal health literacy techniques into routine practice. In contrast, the health improvement advisors reported being more familiar with the techniques and had used them in small ways before the training e.g. use of online quizzes and ‘take home’ messages. They therefore felt more confident to use the techniques in their practice but had little control over making changes to their session slides which were developed by the management team.

*I think the Chunk and Check is just something I’ve never even heard of or done before. So*,* it’s quite a big change to include that in your education sessions. And the slides (that physiotherapist 1 has incorporated into the PowerPoints) have really helped me. So*,* after we’d done the training*,* I can honestly admit that I then did another education session*,* and I didn’t do it. But the new slides do help. They just remind us.* (Physiotherapist 4)

*I feel like we were using a lot of them anyway*,* I don’t know if anybody else thinks that? And I think maybe that’s why it made it easier.* (Health improvement advisor 8)

### Implementing the techniques is challenging in a group setting

For both teams the use of the techniques was limited by delivering their courses to groups of service users rather than one-to-one. They described how difficult it was to use Teach-back in particular in a group setting. The physiotherapists reported how they used the techniques in one-to-one interactions with service users outside the programme in the evaluation e.g. wider respiratory clinics.

*I feel like using Simple Language can work for anyone in any position whether it’s one-to-one or a group. But I struggle sometimes when it’s a group setting to use ‘Chunk and Check.* (Health improvement advisor 10)

*Teach-back’ is more useful on a one-to-one basis really and when you teach inhaler techniques and things like that it’s like ‘right you show me now’. And like [colleague in the focus group] said you do think ‘oh my [goodness] what are they doing!’. So that is really useful. (*Physiotherapist 1)

### More post-training implementation support needed

Implementation support from the health literacy officer included email correspondence and group feedback and advice sessions. Unfortunately, not all the service providers in the evaluation received the support offered due to time constraints. Those that did receive it found it valuable. Suggestions for other kinds of implementation support were made, such as prompt sheets, the health literacy officer observing their practice and telling them where to improve use of the techniques, or time to reflect on their practice as a team.

*Or even after we’ve had the training*,* like shadow us and then give us feedback in terms of like ‘’oh I notice you said this’’*,* ‘’you could have turned it into an open question by saying this’’ would have been really specific and pinpointed where we can get stuff in and what exactly we can do differently*. *Because it does take practice with these things for it to become normal*. (Health improvement adviser 10)

*I think you need prompting because everything’s so busy and*,* like we said*,* it’s not just this project we’re doing*,* is it? How many other things are going on at the same time? We feel like we’re split in many pieces. So*,* I think that’s helpful*,* the prompting and the time to chat and reflect.* (Physiotherapist 3)

### Change in service users’ health literacy levels

In the post-test period there was an improvement in subjective health literacy for service users in both programmes (Table 2). At the end of each programme, service users were more likely to feel they had sufficient information to manage their health, and more likely to feel able to manage their health (*p* < 0.001 for all comparisons). This could indicate the utility of the programmes at improving health literacy, rather than the effect of the verbal health literacy initiative (see Discussion for interpretation of this finding). For the Weight Management Programme, some service users attended programmes led by health-literacy-trained staff, and some service users attended programmes led by staff who had not attended the training (a control group). This allowed for comparison of the initiative with a control. However, numbers of service users in the control group were small (*n* = 6) and the statistical power for this comparison was low. There was no statistically significant difference in change in health literacy levels between initiative and control service users: mean difference=-0.1 (-0.78 to 0.90, *p* = 0.88) for sufficient information and mean difference 0.2 (-0.76 to 0.35, *p* = 0.44) for actively managing health (Table 2).

**Table 2:** Changes in service users’ health literacy levels (two domains of the Health Literacy Questionnaire)

Service	+Sufficient information BASELINE	Sufficient information END	Change	+Actively managing health BASELINE	Actively managing health END	Change	N*
	Mean	Mean	Mean (95% CI)	Mean	Mean	Mean (95% CI)	
Pulmonary Rehabilitation Programme	2.8	3.6	0.8 (0.6–1.1)	2.8	3.3	0.5 (0.3 to 0.7)	40
Weight Management Programme (all)	2.6	3.4	0.8 (0.5–1.2)	2.6	3.1	0.5 (0.3 to 0.8)	20
*Weight Management Programme (attended sessions with health literacy trained staff)*	*2.6*	*3.4*	*0.8* *(0.3–1.3)*	*2.6*	*3.3*	*0.6* *(0.3 to 0.9)*	*14*
*Weight Management Programme (attended sessions with control staff)*	*2.4*	*3.3*	*0.9* *(0.2–1.6)*	*2.5*	*2.9*	*0.4* *(-0.2 to 1.0)*	*6*

+ higher scores indicate higher levels of subjective health literacy

*There was loss to follow-up. 40/49 people completed the Health Literacy Questionnaire at the end of the course for the Pulmonary Rehabilitation Programme, and 20/34 for the Weight Management Programme

### Changes in health outcomes

Primary health outcomes for individual service users who gave consent for their data to be used are shown in Table 3. Secondary outcomes are in supplementary files 9–12. There were improvements in the scores for the Pulmonary Rehabilitation Programme and the Weight Management Programme, showing the impact of the programmes rather than the verbal health literacy training. There was no statistically significant difference between the primary outcome for service users attending the health-literacy-trained group compared with the control group for the Weight Management Programme − 0.2 (-2.3 to 1.9) *p* = 0.84.

**Table 3:** Change in primary outcomes for individual service users giving consent

Service	Baseline	End	Change	*N*	*p*-value
	Mean score	Mean score	Mean change (95% CI)		
Pulmonary Rehabilitation Programme (CRDQ score)					
Dyspnoea Fatigue Emotional function Mastery	2.79 3.65 4.37 4.48	3.37 4.15 4.88 5.10	0.58 (0.11 to 1.10) 0.50 (0.17 to 0.83) 0.51 (0.20 to 0.81) 0.63 (0.27 to 0.99)	41 41 41 41	0.016 0.004 0.002 0.001
Weight Management Programme (Weight in kg)	105.5	102.9	-2.6 (-3.5 to -1.7)	27	< 0.001
*Weight Management Programme (attended sessions with health literacy trained staff)*	*108.8*	*106.2*	*-2.6 (-3.6 to -1.5)*	*20*	*< 0.001*
*Weight Management Programme (attended sessions with control staff)*	*95.6*	*92.9*	*-2.8 (-5.1 to -0.5)*	*7*	*0.024*

Aggregated routine data allowed comparison of outcomes for service users attending programmes in time periods before and after the service providers had attended the verbal health literacy training. For the Pulmonary Rehabilitation Programme, the completion rate of the programme (completed eight or more sessions) pre-training was 57% (97/169) and post-training was 62% (69/111). There was no indication that the verbal health literacy training had improved completion rates (chi-square, *p* = 0.43). There was no indication that the verbal health literacy training had improved service user outcomes (see Table 4 for primary outcome and supplementary file 9 for secondary outcomes). For the Weight Management Programme, the completion rate for the programmes run by health-literacy-trained advisors (completed 9 weeks or more) pre-training was 50% (219/438) and post-training was 56% (215/383) compared with controls pre-training 62% (76/123) and post-training 67% (79/118). There was no indication that the verbal health literacy training had improved service user outcomes. Standard deviations differed largely between time periods and groups (see supplementary files 9–10 for primary and secondary change in outcomes and supplementary files 11–12 for secondary outcomes for those completing the programme only for both services).

**Table 4:** Change in primary outcomes before and after service providers attended the training (based on aggregated routine data for service users completing the programmes)

Service	Primary outcome	Mean change (SD) Before	Mean change (SD) After	Difference in mean changes (95%CI)	*p*-value
Pulmonary Rehabilitation Programme	CRDQ score Dyspnoea Fatigue Emotional function Mastery	0.93 (1.47) 0.74 (1.11) 0.46 (0.98) 0.64 (1.32)	0.71 (1.23) 0.56 (1.15) 0.44 (1.11) 0.54 (1.15)	-0.22 (-0.71 to 0.27) -0.18 (-0.59 to 0.23) -0.02 (-0.39 to 0.36) -0.10 (-0.55 to 0.35)	0.37 0.38 0.92 0.66
N		63	58		
*Weight Management Programme (attended sessions with health literacy trained staff)*	Weight in kg	*-3.16 (0.54)*	*-3.30 (0.51)*	*-0.14 (-0.23 to -0.04)*	*0.006*
		*219*	*215*		
*Weight Management Programme (attended sessions with control staff)*	Weight in kg	*-1.58 (3.06)*	*-3.38 (0.03)*	*-1.80 (-2.4 to -1.1)*	*0.0001*
*N*		*76*	*79*		

## Discussion

### Summary of findings

This was a small mixed methods evaluation of a verbal health literacy initiative. In relation to the proposed theory of change, service providers found the verbal health literacy training useful because they learned new communication techniques. They changed their communication practice, making increased use of techniques when communicating with service users. Both programmes worked with groups of service users rather than on a one-to-one basis. Service providers reported that Teach-back was difficult to use in group settings but was useful in one-to-one sessions in their wider practice. They also found the implementation support from the health literacy officer helpful but wanted more opportunities to meet as teams to reflect on how to improve their use of the new techniques. The health literacy levels of service users improved by the end of their programme. However, this improvement was likely to be due to the programme rather than the verbal health literacy initiative. There was no evidence that the verbal health literacy initiative improved health outcomes for service users in this statistically underpowered analysis.

### Context of other research

A key finding in this study was that it was difficult to apply some of the verbal health literacy techniques in the context of delivering care to groups rather than individual service users. There appears to be a dearth of research that focuses on this aspect of implementing verbal health literacy techniques. However, there is some research that does not support this finding, concluding that incorporating health literacy techniques into information-based group sessions can improve service users’ knowledge [41, 42]. However, in Gharachourlo et al.’s (2018) study they did not describe their health literacy intervention approach [42] and neither Garachourlo et al. (2018) or Hosseinifar et al. (2024) explored service providers’ ability or perceptions of using health literacy strategies when delivering information sessions to groups of service users [41, 42]. This suggests that further research exploring the use of verbal health literacy techniques in a group setting is warranted.

The service providers in this study felt they learnt new communication techniques and changes in practice were observed. Despite this, both teams found Teach-back in particular difficult to implement in the group setting. Cork and White’s (2022) study exploring community pharmacists’ views of a health literacy training initiative found Chunk and Check rather than Teach-back more difficult to implement [19]. It is possible that this was because community pharmacists interacted with service users in a more instructive way and on a one-to-one basis, for example teaching medication use, rather than the information-based group sessions offered in our evaluation. Difficulty implementing Teach-back into practice has been highlighted in two more recent American studies [9, 43]. They found that to overcome issues such as communication habits, time, role constraints and uncertainty on how to use health literacy techniques in practice, changes to the way Teach-back is taught, conceptualised and implemented is needed [9, 43].

In our evaluation, the verbal health literacy initiative for service providers was delivered by a health literacy officer working within the same organisation. The implementation support they offered after the initiative was limited in terms of both resource and capacity related to the role and additional service pressures across the programmes involved. Despite this the support received was valued by the teams. Several studies have explored the use of external health literacy facilitators as well as the use of health literacy champions (clinicians or team members) trained in health literacy techniques, to provide strong leadership and coordinate, support and sustain implementation [9, 20, 21, 44]. It has been proposed that utilising champions from multiple levels within an organisation who work together in a coordinated way, such as a ‘community of practice’, as well as training existing staff to deliver and sustain initiatives may be more effective than solo champions [9, 21, 44]. This bottom-up, rather than top-down approach, may give staff the autonomy and confidence to make and sustain changes at a practice level [9, 21, 44]. However, in an ideal world a combination of top-down, such as a health literacy officer, and bottom-up approaches should be considered to optimise an organisation’s ability to embed health literacy into healthcare practice [20].

The implementation support provided by the health literacy officer in our evaluation was developed specifically for the context of this study. It may be that further emphasis on developing this part of the intervention could facilitate effectiveness. In fact, the service providers in this evaluation suggested alternative implementation support strategies. These included prompt sheets and having regular protected time to review and reflect on how they were using the verbal health literacy techniques in their practice. In the wider literature, implementation support methods include the use of flyers [44], ‘lunch and learn’ sessions to discuss what is and is not working [9, 44], five minute booster sessions during breaks [9, 44], weekly emails [9], staff induction [44] and standardised document templates to record the use of techniques such as Teach-back in clinical notes [44]. A range of support methods that can be easily incorporated into busy clinical practice could be considered in future initiatives, including the development of health literacy champions within service user facing teams to support and embed implementation, in any future delivery of a verbal health literacy initiative.

Despite increasing research into verbal health literacy initiatives, few studies have evaluated initiatives by assessing service providers’ health literacy knowledge and the ability to apply that knowledge in practice. There are validated measures to do this [45] and future research could apply these measures in organisations attempting to improve organisational health literacy.

### Strengths and limitations

This was a mixed methods evaluation. It used a range of qualitative and quantitative methods to address the acceptability and effectiveness of a new initiative in a real-world setting. This study had three strengths. First, the comparison of two different health programmes with different protocols, using different outcome measures, serving service users with different health issues. Second, the measurement of service user health outcomes using a controlled before and after design for one of the programmes implementing the new initiative. Third, the inclusion of results for both service providers and service users. There were eight limitations. First, it was a small evaluation in terms of the small numbers of staff trained and the small numbers of service users in some of the analyses. Second, the controlled before and after design was not possible for one of the programmes. Instead, this programme relied on a before and after design which is lower in the hierarchy of evidence of effectiveness. Third, the consent process to include data from individual service users was challenging. Physiotherapists worked hard to gain consent and did well in recruiting service users. The electronic approach to recruitment for the Weight Management Programme yielded a very low consent rate. Small numbers limited the statistical power to detect changes in individual service user data. The included individual data may not be representative of service users. More educated or affluent service users may have completed the consent process. There was no data to explore this concern. Fourth, there was drop out of service users over time, with some service users not completing their end of course outcome measures. Fifth, the health outcomes analysis was under powered. Sixth, the views of service users were not collected so it is not known if the initiative enabled clear conversations from their perspective. Seventh, the evaluation paid no attention to contamination whereby service providers who were not trained had the potential to informally learn techniques from their trained colleagues. Eighth, standard deviations differed largely between time periods and groups when comparing outcomes in the aggregate data. This could indicate some inaccuracies in the routine data.

### Implications

This verbal health literacy initiative did not improve service user outcomes. While this was one of the main goals of this study it is likely that this type of communication intervention will need other supportive factors, both organisational and professional peer support, to demonstrate a direct effect on health outcome. The service providers however valued the training and could see the potential of the learnt techniques to improve communication with patients. Implications for a future verbal health literacy initiative include having a more intense approach to supporting implementation of the verbal health literacy techniques. It may also be necessary to include a managerial component because some of the service providers in our study had to use materials developed by their managers. To fully apply their learning, they would need to have sufficient control over making changes to those materials. Also, any similar initiative might fare better in the context of one-to-one consultations, although further adaptation of the training to group delivery would also be useful.

There are also some implications for evaluating these kinds of initiatives. Future mixed methods evaluations should include controlled before and after measurement of patient reported outcomes measures with larger sample sizes. This would allow a better understanding of the impact these kinds of verbal health literacy initiatives can have on health outcomes. However, if this type of comparison relies on service user consent, it will likely lead to small, biased samples. The use of aggregated routine data could offer a better option. Even in the context of a robust design, attributing outcomes to verbal health literacy initiatives faces the challenge of differentiating between improvements in health literacy due to the pulmonary rehabilitation and weight management programmes and the verbal health literacy initiative, along with other sources of bias such as selection.

An interesting aspect of our evaluation was that both services offered group-based rather than on-to-one care. Experts developing future health literacy initiatives could consider what adaptations are needed for these group-based contexts.

## Conclusions

In this small evaluation, the verbal health literacy initiative had potential. It was appreciated by service providers and, from the perspective of service providers, enabled clearer conversations with service users. The communication techniques within the initiative may be easier to apply in one-to-one consultations with service users than in the group settings evaluated here. More focus on supporting implementation of techniques in practice, and opportunity for team reflection, might improve the potential effectiveness of the initiative. Future research should focus on a more intense initiative and use controlled before and after designs on larger samples to measure the effect on service users’ health literacy levels and health and wellbeing outcomes.

## Supplementary Information

Below is the link to the electronic supplementary material.

Supplementary Material 1: Supplementary. file 1- The verbal health literacy training schedule

Supplementary Material 2: Supplementary. file 2 - Evaluation components timeline

Supplementary Material 3: Supplementary. file 3- Staff pre training survey

Supplementary Material 4: Supplementary. file 4- Staff post training survey

Supplementary Material 5: Supplementary. file 5 – Staff follow up post training survey

Supplementary Material 6: Supplementary file 6- Table S6. Service provider -How useful was the training

Supplementary Material 7: Supplementary file 7- Table S7. Service provider confidence to use the Verbal Health Literacy techniques

Supplementary Material 8: Supplementary file 8- Table S8. Service provider frequency of use for the verbal health literacy techniques in practice

Supplementary Material 9: Supplementary file 9. Table S9. Change in primary and secondary outcomes for individual service users in Pulmonary Rehabilitation Programme

Supplementary Material 10: Supplementary file 10. Table S10. Change in secondary outcome of wellbeing for individual service users in Weight Management Programme

Supplementary Material 11: Supplementary file 11. Table S11. Pulmonary Rehabilitation Programme secondary health outcomes (those completing the programme only)

Supplementary Material 12: Supplementary file 12. Table S12. Weight Management Programme wellbeing secondary outcome (those completing the programme only)

## Data Availability

The datasets used and/or analysed during the current study are available from the corresponding author on reasonable request.
