# Supplementary material for: Clear Conversations: a mixed methods evaluation of a verbal health literacy initiative for health service providers
Source: BMC Health Serv Res. 2026 May 9;26:905. doi: 10.1186/s12913-026-14684-y (PMC13326052; doi:10.1186/s12913-026-14684-y)
Supplement: Supplementary file 1 — Supplementary Material 1: Supplementary. file 1- The verbal health literacy training schedule [file 12913_2026_14684_MOESM1_ESM.pdf]

Appendix 1 The Verbal Health Literacy Training schedule

Time	Duration	Content / Slides	Format
00:00–00:05	5 mins	Welcome, housekeeping, aims	Intro
00:05–00:10	5 minutes	Health literacy icebreaker – difficult words	Menti
00:10–00:20	10 mins	“Draw the monster” activity	Interactive + discussion
00:20–00:35	15 mins	What is health literacy? Why does it matter?	Input + discussion
00:35–00:40	5 mins	What can we do? Focus on verbal communication	Discussion
00:40–00:55	15 mins	Using simple language + short video	Input+ discussion
00:55–01:05	10 mins	Simple language card-pairing activity	Group activity
**01:05–01:10**	**5 mins**	**Comfort break**	—
01:10–01:25	15 mins	Teachback – explanation + video discussion	Input
01:25–01:40	15 mins	Teachback practice in small groups	Practice
01:40–01:55	15 mins	Chunk & Check + monster revisit	Interactive
01:55–02:00	5 mins	Key takeaways, evaluation, close	Plenary
