# Supplementary figures and images for: Clear Conversations: a mixed methods evaluation of a verbal health literacy initiative for health service providers
Source: BMC Health Serv Res. 2026 May 9;26:905. doi: 10.1186/s12913-026-14684-y (PMC13326052; doi:10.1186/s12913-026-14684-y)

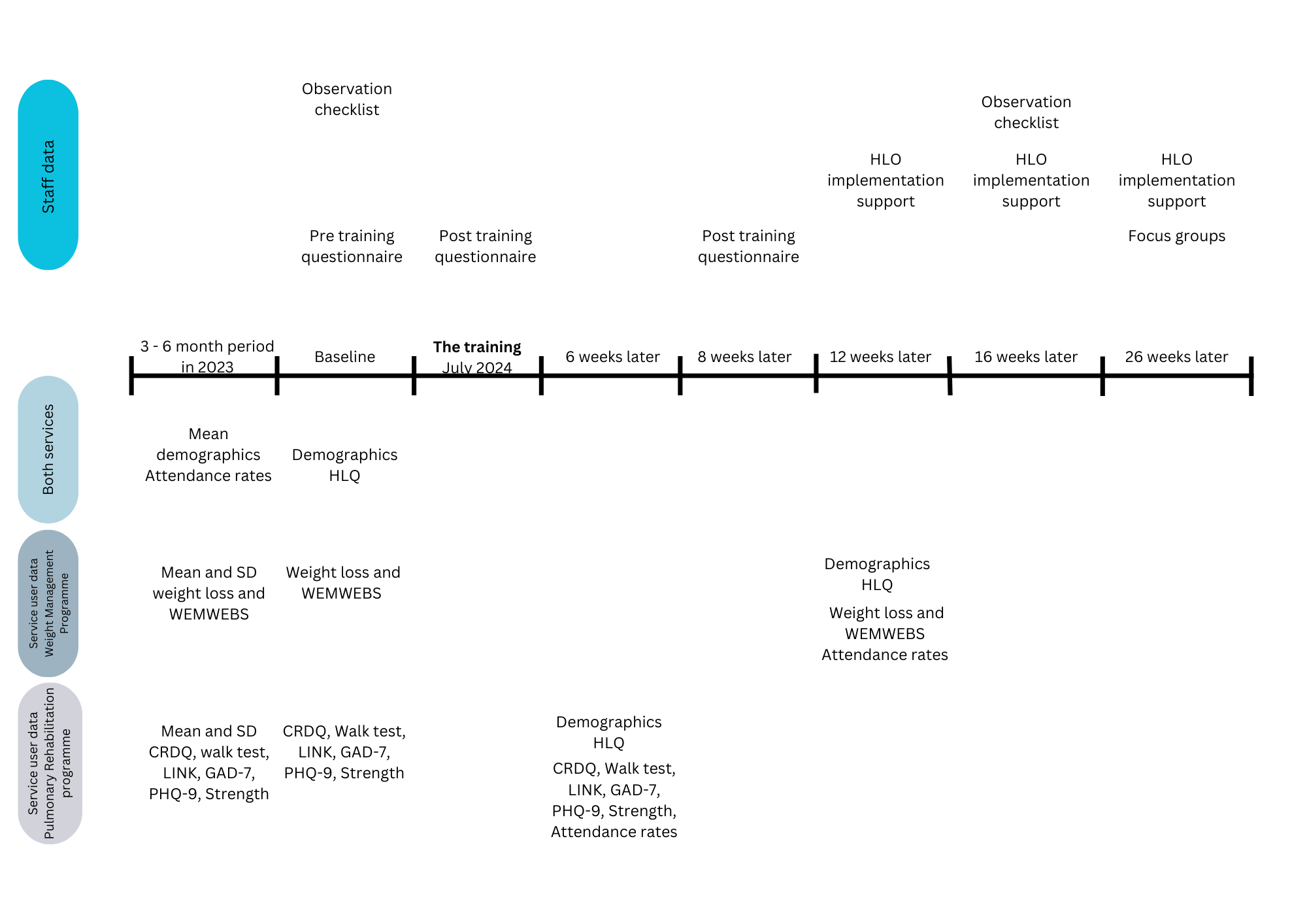

Supplement: Supplementary file 2 — Supplementary Material 2: Supplementary. file 2 - Evaluation components timeline [file 12913_2026_14684_MOESM2_ESM.png]
