# Supplementary material for: Clear Conversations: a mixed methods evaluation of a verbal health literacy initiative for health service providers
Source: BMC Health Serv Res. 2026 May 9;26:905. doi: 10.1186/s12913-026-14684-y (PMC13326052; doi:10.1186/s12913-026-14684-y)
Supplement: Supplementary file 3 — Supplementary Material 3: Supplementary. file 3- Staff pre training survey [file 12913_2026_14684_MOESM3_ESM.pdf]

Verbal Health Literacy Training
Pre-Course Evaluation

Thank you for taking the time to complete this questionnaire.

It should take you 5 minutes to complete.

We are keen to understand what impact the Verbal Health Literacy training has on your verbal communication.

We will ask you to complete a questionnaire:

- Immediately before you attend the Verbal Health Literacy training.
- Immediately after the training.
- Two months after the training.

* Required

1. We would like to include your answers from this questionnaire in The Clear Conversations Research Project.

   The Clear Conversations Research Project is being run by The University of Sheffield. They are working in partnership with Derbyshire Community Health Services and Derbyshire County Council.

   The research project aims to improve health workers communication skills.

We want to see if this can improve service user health outcomes and reduce health inequalities.

The information you provide us will help us understand and improve communication skills training in the future.

We will keep your views anonymous and confidential.

·We will ask you to create a 'code name' and to use the same one for each questionnaire you complete.

· This is so you are not identifiable in any of the data we collect.

You do not have to agree for your answers to be part of the research project. You will still receive the training and we will still ask you to complete the questionnaire.

If you would like more information about The Clear Conversations Research project please contact the Lead Researcher, Cheryl Grindell at: c.a.grindell@sheffield.ac.uk

The Clear Conversations Research Project has received ethical approval from the HRA/NHS ethics committee [insert ethics number here].

○ **I do** consent to my anonymised answers in this questionnaire to be used in the Clear Conversations Research project.

○ **I do not** consent to my anonymised answers in this questionnaire to be used in the Clear Conversations Research project.

2. **Code Name**: Please create a code name.

   Put the first three letters of the road you live on followed by the day of your birthday.

   For example, if you live on **Ham**ble Close and you were born **12**th Sept, your code name would be **HAM12**.

   Please use the same code each time you complete an evaluation. This will help us to compare your answers. *

   Enter your answer

3. **Organisation:** *

- ◯ CRH Chesterfield Royal Hospital
- ◯ DCHS Derbyshire Community Health Services
- ◯ Derby City Council
- ◯ Derbyshire County Council
- ◯ DHcFT Derbyshire Healthcare NHS Foundation Trust (Mental Health)
- ◯ ICB - NHS Derby and Derbyshire Integrated Care Board
- ◯ Primary Care
- ◯ UHDB University Hospitals Derby and Burton
- ◯ Voluntary, Community or Social Enterprise Sector
- ◯ Other

4. **Role/Profession:**
Please select the role closest to the job you do. *

- ◯ Admin/Clerical
- ◯ Allied Health Professional
- ◯ Local Authority/Local Government
- ◯ Management/Leadership
- ◯ Medical/Doctor
- ◯ Nursing
- ◯ Pharmacy

- Psychology
- Public Health
- Social Care Provider
- Social/Community Prescribing
- Social Worker
- Voluntary, Community or Social Enterprise Sector Worker

5. Date of training you are attending: *

Please input date (dd/MM/yyyy)

6. Have you previously completed any Health Literacy training ? *

- [ ] Health Literacy Awareness E-Learning Module
- [ ] Health Literacy Awareness Face to Face Session
- [ ] Health Literacy Awareness Virtual Session
- [ ] Health Literate Organisation Workshop
- [ ] Not attended any Health Literacy training
- [ ] Other

7. Do you have any learning or additional support requirements? *

- Yes
- No

Never give out your password. Report abuse

Microsoft 365

This content is created by the owner of the form. The data you submit will be sent to the form owner. Microsoft is not responsible for the privacy or security practices of its customers, including those of this form owner. Never give out your password.

Microsoft Forms | AI-Powered surveys, quizzes and polls Create my own form

Privacy and cookies | Terms of use
