# Supplementary material for: Clear Conversations: a mixed methods evaluation of a verbal health literacy initiative for health service providers
Source: BMC Health Serv Res. 2026 May 9;26:905. doi: 10.1186/s12913-026-14684-y (PMC13326052; doi:10.1186/s12913-026-14684-y)
Supplement: Supplementary file 5 — Supplementary Material 5: Supplementary. file 5 – Staff follow up post training survey [file 12913_2026_14684_MOESM5_ESM.pdf]

Verbal Health Literacy Training Follow Up Post-Course Evaluation

Thank you for taking the time to complete this questionnaire.

It should take you 5 minutes to complete.

We are keen to understand what impact the Verbal Health Literacy training has on your verbal communication.

We will ask you to complete a questionnaire:

o Immediately before you attend the Verbal Health Literacy training.

o Immediately after the training.

o Two months after the training.

* Required

Clear Conversations Research Project

Will you consent to your answers being used in our research project?

1. We would like to include your answers from this questionnaire in The Clear Conversations Research Project.

   The Clear Conversations Research Project is being run by The University of Sheffield. They are working in partnership with Derbyshire Community Health Services and Derbyshire County Council.

   The research project aims to improve health workers communication skills.

   We want to see if this can improve service user health outcomes and reduce health inequalities.

   The information you provide us will help us understand and improve communication skills training in the future.

   We will keep your views anonymous and confidential.

   · We will ask you to create a 'code name' and to use the same one for each questionnaire you complete.

   · This is so you are not identifiable in any of the data we collect.

   You do not have to agree for your answers to be part of the research project. You will still receive the training and we will still ask you to complete the questionnaire.

   If you would like more information about The Clear Conversations Research project please contact the Lead Researcher, Cheryl Grindell at: c.a.grindell@sheffield.ac.uk

   The Clear Conversations Research Project has received ethical approval from the HRA/NHS ethics committee [insert ethics number here].

   ◯ **I do** consent to my anonymised answers in this questionnaire to be used in the Clear Conversations Research project.

   ◯ **I do not** consent to my anonymised answers in this questionnaire to be used in the Clear Conversations Research project.

2. **Code Name**: Please create a code name.

   Put the first three letters of the road you live on followed by the day of your birthday.

   For example, if you live on **Ham**ble Close and you were born **12**th Sept, your code name would be **HAM12**.

   Please use the same code each time you complete an evaluation. This will help us to compare your answers. *

3. **Organisation:**

   - ◯ CRH Chesterfield Royal Hospital
   - ◯ DCHS Derbyshire Community Health Services
   - ◯ Derby City Council
   - ◯ Derbyshire County Council
   - ◯ DHcFT (Derbyshire Healthcare NHS Foundation Trust (Mental Health)
   - ◯ ICB - NHS Derby and Derbyshire Integrated Care Board
   - ◯ Primary Care
   - ◯ UHBD University Hospitals Derby and Burton
   - ◯ Voluntary, Community or Social Enterprise Sector Worker
   - ◯ Other

4. **Role/Profession:**
Please select the role closest to the job you do.

- ◯ Admin/Clerical
- ◯ Allied Health Professional
- ◯ Local Authority/Local Government
- ◯ Management/Leadership
- ◯ Medical/Doctor
- ◯ Nursing
- ◯ Pharmacy
- ◯ Psychology
- ◯ Public Health
- ◯ Social Care Provider
- ◯ Social/Community Prescribing
- ◯ Social Worker
- ◯ Voluntary, Community or Social Enterprise Sector Worker

5. Date of training you attended: *

I

6. On a scale from 1 to 5, how would you rate your **knowledge** of these verbal health literacy techniques? *

	1. Poor	2. less than adequate	3. Adequate	4. Good
The use of simple language	◯	◯	◯	◯
Teachback	◯	◯	◯	◯
Chunk and Check	◯	◯	◯	◯

7. On a scale from 1 to 5, how would you rate your **confidence** in using these verbal health literacy techniques in your conversations: *

	1. Poor	2. Less than adequate	3. Adequate	4. Good	5.
Using simple language	◯	◯	◯	◯	
Using teachback	◯	◯	◯	◯	
Using chunk and check	◯	◯	◯	◯	

8. Please rate how much you are able to use the following in your conversations
(please evaluate your level honestly, rather than what you believe it should be). *

	Not at all	Rarely	Sometimes	Often
I use words that can be easily understood	◯	◯	◯	◯
I use acronyms and jargon (e.g. MDT)	◯	◯	◯	◯
I ask people "Do you Understand?"	◯	◯	◯	◯
I take responsibility for explaining information clearly and use teachback to check if people understand	◯	◯	◯	◯
I give people a lot of information all in one go and then ask if they understand it at the end.	◯	◯	◯	◯
I break down information into small chunks and check it is clear at each point.	◯	◯	◯	◯

9. I have an **opportunity** to use verbal health literacy techniques in my day to day practice. *

◯ Never

◯ Rarely

◯ Sometimes

◯ Very Often

◯ Always

◯ Not sure

10. What gets in the way of you using verbal health literacy techniques in your conversations? Select all that apply:

- [ ] Lack of knowledge
- [ ] Lack of confidence
- [ ] Limited time
- [ ] Needing to complete a lot of paperwork
- [ ] Other

11. In your role, think about a conversation you might have with people where you need to share some new information.

    What might you say to check that this information has been understood?

    Please write one or two sentences below: *

12. On a scale of 1-5 how **confident** would you feel in a scenario where you need to check that you have explained new information clearly?

(1= not at all confident, 5 = very confident) *

◯ **1**

◯ **2**

◯ **3**

◯ **4**

◯ **5**

13. Would you be interested in any of the following to support you in using verbal health literacy techniques in your role?

	Not interested	Quite interested	Very Interested
Joining an online session to practice using verbal health literacy techniques	◯	◯	◯
Watching short videos/webinars which demonstrate verbal health literacy techniques	◯	◯	◯
Other	◯	◯	◯

14. **Any other comments?**
This could include:

- Suggestions you have for activities/resources that would support you to continue using verbal health literacy techniques in your role.
- Any examples of how you have been able to use some of the techniques so far.
- Any further reflections you've had on how the training could be improved.

This content is neither created nor endorsed by Microsoft. The data you submit will be sent to the form owner.

Microsoft Forms
