# Supplementary material for: Clear Conversations: a mixed methods evaluation of a verbal health literacy initiative for health service providers
Source: BMC Health Serv Res. 2026 May 9;26:905. doi: 10.1186/s12913-026-14684-y (PMC13326052; doi:10.1186/s12913-026-14684-y)
Supplement: Supplementary file 6 — Supplementary Material 6: Supplementary file 6- Table S6. Service provider -How useful was the training [file 12913_2026_14684_MOESM6_ESM.docx]

**Table S6. How useful was the training**

|  | **All service providers**  **%** | **Service providers in our study**  **%** | **Pulmonary Rehab Programme service providers**  **%** | **Weight Management Programme service providers**  **%** |
| --- | --- | --- | --- | --- |
|  | **n=69** | **n=11** | **n=5** | **n=6** |
| **How useful did you find the training?**  1 Not at all useful  2 Quite useful  3 Useful  4 Very useful | 2.9  2.9  26.1  68.1 | 9.1  0  27.3  63.6 | 0  0  0  100 | 16.7  0  50  33.3 |
