# Supplementary material for: Clear Conversations: a mixed methods evaluation of a verbal health literacy initiative for health service providers
Source: BMC Health Serv Res. 2026 May 9;26:905. doi: 10.1186/s12913-026-14684-y (PMC13326052; doi:10.1186/s12913-026-14684-y)
Supplement: Supplementary file 7 — Supplementary Material 7: Supplementary file 7- Table S7. Service provider confidence to use the Verbal Health Literacy techniques [file 12913_2026_14684_MOESM7_ESM.docx]

**Table S7. Service provider confidence to use the verbal health literacy techniques**

| **Question** | **All service providers**  **%** | | | **Service providers in our study**  **%** | | | **Pulmonary Rehab Programme service providers**  **%** | | | **Weight Management Programme service providers**  **%** | | |
| --- | --- | --- | --- | --- | --- | --- | --- | --- | --- | --- | --- | --- |
|  | **n=110** | **n=69** | **n=23** | **n=11** | **n=11** | **n=7** | **n=5** | **n=5** | **n=3** | **n=6** | **n=6** | **n=4** |
| On a scale of 1-5 (1 Poor – 5 Excellent) how would you rate your confidence to use the following: | **Pre** | **Post** | **Follow Up** | **Pre** | **Post** | **Follow Up** | **Pre** | **Post** | **Follow Up** | **Pre** | **Post** | **Follow Up** |
| **Teach-back**  1 Poor  2 Less than adequate  3 Adequate  4 Good  5 Excellent | 14.5  36.4  32.7  14.5  1.8 | 0  1.4  14.5  59.4  24.6 | 0  0  21.7  60.9  17.4 | 18.2  45.4  36.4  0  0 | 0  0  18.2  72.7  9.1 | 0  0  28.6  57.1  14.3 | 20  40  40  0  0 | 0  0  20  80  0 | 0  0  66.7  0  33.3 | 0  16.7  66.7  16.7  0 | 0  0  16.7  66.7  16.7 | 0  0  0  100  0 |
| **Chunk and Check**  1 Poor  2 Less than adequate  3 Adequate  4 Good  5 Excellent | 12.7  32.7  36.4  16.4  1.8 | 0  1.4  14.5  53.6  30.4 | 0  0  21.7  60.9  17.4 | 27.3  45.5  27.3  0  0 | 0  0  18.2  72.7  9.1 | 0  0  42.9  57.1  0 | 40  40  20  0  0 | 0  0  20  80  0 | 0  0  66.7  33.3  0 | 16.7  50  33.3  0  0 | 0  0  16.7  66.7  16.7 | 0  0  25  75  0 |
| **Simple language**  1 Poor  2 Less than adequate  3 Adequate  4 Good  5 Excellent | 5.5  14.5  36.4  39.1  4.5 | 0  0  7.2  56.5  36.2 | 0  0  8.7  60.9  30.4 | 0  9.1  54.5  36.4  0 | 0  0  0  63.6  36.4 | 0  0  0  85.7  14.3 | 0  0  40  60  0 | 0  0  0  100  0 | 0  0  0  66.7  33.3 | 16.7  50  33.3  0  0 | 0  0  0  33.3  66.7 | 0  0  0  100  0 |
