# Supplementary material for: Clear Conversations: a mixed methods evaluation of a verbal health literacy initiative for health service providers
Source: BMC Health Serv Res. 2026 May 9;26:905. doi: 10.1186/s12913-026-14684-y (PMC13326052; doi:10.1186/s12913-026-14684-y)
Supplement: Supplementary file 8 — Supplementary Material 8: Supplementary file 8- Table S8. Service provider frequency of use for the verbal health literacy techniques in practice [file 12913_2026_14684_MOESM8_ESM.docx]

**Table S8 Service provider reported frequency of use of the verbal health literacy techniques in practice**

| **Question** | **All service providers**  **%** | | | **In our study**  **%** | | | **Pulmonary Rehab Programme**  **%** | | | **Weight Management Programme**  **%** | | |
| --- | --- | --- | --- | --- | --- | --- | --- | --- | --- | --- | --- | --- |
| On a scale of 0-4 (0 not at all – 4 very often) in your current practice how often **do you use (pre)**/**plan to use (post)/are able to use (follow up)** these approaches in your conversations? | **Pre**  **n=110** | **Post**  **n=58** | **Follow Up**  **n=15** | **Pre**  **n=11** | **Post**  **n=11** | **Follow Up**  **n=7** | **Pre**  **n=5** | **Post**  **n=5** | **Follow Up**  **n=3** | **Pre**  **n=6** | **Post**  **n=6** | **Follow Up**  **n=4** |
| **Teach-back**  1 Not at all  2 Rarely  3 Sometimes  4 Often  5 Very often | 6.4  16.4  37.3  34.5  5.5 | 0  0  7.2  37.7  55.1 | 0  8.7  4.3  60.9  26.1 | 18.2  18.2  54.5  9.1  0 | 0  0  18.2  36.4  45.4 | 0  0  14.3  85.7  0 | 20  40  40  0  0 | 0  0  20  40  40 | 0  0  0  100  0 | 16.7  0  66.7  16.7  0 | 0  0  16.7  33.3  50 | 0  0  25  75  0 |
| **Chunk and Check**  1 Not at all  2 Rarely  3 Sometimes  4 Often  5 Very often | 1.8  9.1  40.9  44.5  3.6 | 0  1.4  5.8  36.2  56.5 | 0  4.3  13.0  47.8  34.8 | 9.1  9.1  54.5  27.3  0 | 0  9.1  0  45.5  45.5 | 0  0  42.9  57.1  0 | 20  0  60  20  0 | 0  0  0  40  60 | 0  0  33.3  66.7  0 | 0  16.7  50  33.3  0 | 0  16.7  0  50  33.3 | 0  0  50  50  0 |
| **Simple language**  1 Not at all  2 Rarely  3 Sometimes  4 Often  5 Very often | 0.9  3.6  15.5  52.7  27.3 | 0  0  4.3  39.1  56.5 | 0  0  56.5  43.5  0 | 0  0  9.1  72.7  18.2 | 0  0  0  45.5  54.5 | 0  0  0  57.1  42.9 | 0  0  0  80  20 | 0  0  0  80  20 | 0  0  66.7  33.3  0 | 0  0  16.7  66.7  16.7 | 0  0  0  16.7  83.3 | 0  0  50  50  0 |
