# Supplementary material for: Clear Conversations: a mixed methods evaluation of a verbal health literacy initiative for health service providers
Source: BMC Health Serv Res. 2026 May 9;26:905. doi: 10.1186/s12913-026-14684-y (PMC13326052; doi:10.1186/s12913-026-14684-y)
Supplement: Supplementary file 9 — Supplementary Material 9: Supplementary file 9. Table S9. Change in primary and secondary outcomes for individual service users in Pulmonary Rehabilitation Programme [file 12913_2026_14684_MOESM9_ESM.docx]

**Table S9 Change in primary and secondary outcomes for individual service users in Pulmonary Rehabilitation Programme**

| **Service** | **BASELINE** | **END** | **CHANGE** | **N for paired comparison** | **p-value** |
| --- | --- | --- | --- | --- | --- |
|  | **Mean** | **Mean** | **Mean (95% CI)** |  |  |
| **Pulmonary Rehabilitation Programme (CRDQ score)**  **Dysnoea**  **Fatigue**  **Emotional function**  **Mastery** | 2.79  3.65  4.37  4.48 | 3.37  4.15  4.88  5.10 | 0.58 (0.11 to 1.10)  0.50 (0.17 to 0.83)  0.51 (0.20 to 0.81)  0.63 (0.27 to 0.99) | 41  41  41  41 | 0.016  0.004  0.002  0.001 |
| **Incremental shuttle walk test** | 227.6 | 289.2 | 61.6 (43.9 to 79.4) | 36 | <0.001 |
| **LINQ** | 8.66 | 3.66 | -5.0 (-6.2 to -3.8) | 40 | <0.001 |
| **GAD** | 5.22 | 4.05 | -1.2 (-2.5 to 0.11) | 40 | 0.073 |
| **PHQ** | 6.77 | 6.08 | -0.7 (-1.8 to 0.4) | 38 | 0.211 |
| **Grip** | 28.5 | 30.3 | 1.7 (0.4 to 3.1) | 35 | 0.013 |
