# Supplementary material for: Clear Conversations: a mixed methods evaluation of a verbal health literacy initiative for health service providers
Source: BMC Health Serv Res. 2026 May 9;26:905. doi: 10.1186/s12913-026-14684-y (PMC13326052; doi:10.1186/s12913-026-14684-y)
Supplement: Supplementary file 10 — Supplementary Material 10: Supplementary file 10. Table S10. Change in secondary outcome of wellbeing for individual service users in Weight Management Programme [file 12913_2026_14684_MOESM10_ESM.docx]

**Table S10 Change in secondary outcome of well-being for individual service users in Weight Management Programme**

|  | **BASELINE** | **END** | **MEAN CHANGE (95% CI)** | **N** | **p-value** |
| --- | --- | --- | --- | --- | --- |
| **Whole group** | 27.1 | 27.2 | 0.2 (-2.2 to 2.5) | 12* | p=0.88 |
| **Intervention group** | 28.6 | 27.8 | -0.9 (-2.8 to 1.0) | 8 | p=0.32 |
| **Control group** | 24.0 | 26.2 | 2.2 (-6.3 to 10.8) | 4 | p=0.46 |

*large number of missing values for measurements of well-being at the end of the programme

+difference between intervention and control group 3.1 (-1.7 to 7.9) p=0.18
