# Supplementary material for: Clear Conversations: a mixed methods evaluation of a verbal health literacy initiative for health service providers
Source: BMC Health Serv Res. 2026 May 9;26:905. doi: 10.1186/s12913-026-14684-y (PMC13326052; doi:10.1186/s12913-026-14684-y)
Supplement: Supplementary file 11 — Supplementary Material 11: Supplementary file 11. Table S11. Pulmonary Rehabilitation Programme secondary health outcomes (those completing the programme only) [file 12913_2026_14684_MOESM11_ESM.docx]

**Table S11 Pulmonary Rehabilitation Programme secondary health outcomes (those completing the programme only)**

|  | **Walking distance** | **LINQ** | **GAD-7** | **PHQ-9** | **Grip** |
| --- | --- | --- | --- | --- | --- |
| **Mean change before training (SD)** | 58.2 (79.2)  N=83 | -4.8 (3.8)  N=58 | -1.2 (4.3)  N=82 | -1.2 (4.5)  N=82 | 2.2 (6.5)  N=81 |
| **Mean change after training (SD)** | 55.9 (61.3)  N=58 | -4.7 (3.5)  N=49 | -1.8 (4.0)  N=56 | -1.7 (4.3)  N=55 | 2.3 (5.0)  N=56 |
| **Change in means (95% CI)** | -2.3 (-26.8 to 22.2) | 0.1 (-1.3 to 1.5) | -0.6 (-2.0 to 0.8) | -0.5 (-2.0 to 1.0) | 0.1 (-1.9 to 2.1) |
| **p-values** | 0.85 | 0.89 | 0.40 | 0.52 | 0.92 |
