# Supplementary material for: Clear Conversations: a mixed methods evaluation of a verbal health literacy initiative for health service providers
Source: BMC Health Serv Res. 2026 May 9;26:905. doi: 10.1186/s12913-026-14684-y (PMC13326052; doi:10.1186/s12913-026-14684-y)
Supplement: Supplementary file 12 — Supplementary Material 12: Supplementary file 12. Table S12. Weight Management Programme wellbeing secondary outcome (those completing the programme only) [file 12913_2026_14684_MOESM12_ESM.docx]

**Table S12 Weight Management Programme Wellbeing secondary outcome (those completing the programme only)**

| **Warwick and Edinburgh mental wellbeing scale (SF)** | **BEFORE** | **AFTER** |  | **p-value** |
| --- | --- | --- | --- | --- |
| **Change in means intervention** | 1.84 (1.34)  N=219 | 1.36 (0.06)  N=215 | -0.48 (-0.66 to -0.30) | 0.001 |
| **Change in means control** | 3.50 (1.53)  N=76 | 3.50 (0.21)  N=79 | 0.0 (-0.34 to 0.34) | 1.00 |
